# Influence of Material Optical Properties in Direct ToF LiDAR Optical Tactile Sensing: Comprehensive Evaluation

**DOI:** 10.3390/ma18143287

**Published:** 2025-07-11

**Authors:** Ilze Aulika, Andrejs Ogurcovs, Meldra Kemere, Arturs Bundulis, Jelena Butikova, Karlis Kundzins, Emmanuel Bacher, Martin Laurenzis, Stephane Schertzer, Julija Stopar, Ales Zore, Roman Kamnik

**Affiliations:** 1 Institute of Solid State Physics, University of Latvia, Kengaraga iela 8, LV-1063 Riga, Latvia; andrejs.ogurcovs@cfi.lu.lv (A.O.); meldra.kemere@cfi.lu.lv (M.K.); arturs.bundulis@cfi.lu.lv (A.B.); jelena.butikova@cfi.lu.lv (J.B.); karlis.kundzins@cfi.lu.lv (K.K.); 2 French-German Research Institute of Saint-Louis, 5 rue du General Cassagnou, 68301 Saint-Louis Cedex, France; emmanuel.bacher@isl.eu (E.B.); martin.laurenzis@isl.eu (M.L.); stephane.schertzer@isl.eu (S.S.); 3 Faculty of Electrical Engineering, University of Ljubljana, Trzaska 25, 1000 Ljubljana, Slovenia; julija.stopar@fe.uni-lj.si (J.S.); ales.zore@fe.uni-lj.si (A.Z.); roman.kamnik@fe.uni-lj.si (R.K.)

**Keywords:** optical tactile sensing, time-of-flight (ToF) LiDAR, frustrated total internal reflection (FTIR), waveguide materials, light scattering, diffuse reflectance, refractive index, contact detection, proximity sensing, silicone resin, 3D-printed polymer materials, acrylic glass

## Abstract

Optical tactile sensing is gaining traction as a foundational technology in collaborative and human-interactive robotics, where reliable touch and pressure feedback are critical. Traditional systems based on total internal reflection (TIR) and frustrated TIR (FTIR) often require complex infrared setups and lack adaptability to curved or flexible surfaces. To overcome these limitations, we developed OptoSkin—a novel tactile platform leveraging direct time-of-flight (ToF) LiDAR principles for robust contact and pressure detection. In this extended study, we systematically evaluate how key optical properties of waveguide materials affect ToF signal behavior and sensing fidelity. We examine a diverse set of materials, characterized by varying light transmission (82–92)%, scattering coefficients (0.02–1.1) cm^−1^, diffuse reflectance (0.17–7.40)%, and refractive indices 1.398–1.537 at the ToF emitter wavelength of 940 nm. Through systematic evaluation, we demonstrate that controlled light scattering within the material significantly enhances ToF signal quality for both direct touch and near-proximity sensing. These findings underscore the critical role of material selection in designing efficient, low-cost, and geometry-independent optical tactile systems.

## 1. Introduction

### 1.1. Background on Optical Tactile Sensing

Optical tactile sensing is an advancing field critical for robotics, human–machine interaction, and flexible electronic interfaces. Early efforts (1990–2015) centered on rigid devices embedding discrete optical paths, often based on total internal reflection (TIR), waveguide deformation, or light modulation to capture tactile information. Maekawa et al. [1] pioneered a finger-shaped optical waveguide sensor detecting contact and surface normals via disrupted TIR. Begej [2] later extended this with planar and fingertip-shaped FTIR sensors using CCD cameras and fiber bundles to generate spatial force maps. Heo et al. [3] utilized optical fiber Bragg gratings and microbending sensors in elastomers to convert pressure to optical signal variations. Simultaneously, vision-based sensors began to emerge. Ito et al. [4] proposed a method using RGB image data from a fluid-filled elastomer to recover object surface geometry, while Lepora and Ward-Cherrier [5] developed the TacTip system, achieving superresolution contact localization by tracking internal marker displacements through Bayesian inference. Graphene-integrated sensors, like that of Kim et al. [6], introduced tunable light absorption for pressure detection in waveguides, overcoming limitations of traditional directional couplers. These foundational systems, though often bulky or limited in adaptability, established the core principles of optical tactile sensing: integrating optical structures into compliant substrates and extracting high-fidelity force or shape information.

Since 2015, research in optical tactile sensing has shifted toward miniaturized, flexible, and multimodal platforms—spanning from fiber-optic sensors for curved surgical instruments to graphene-enhanced waveguide modules for dynamic pressure measurement. Examples include compact optical waveguide sensors for minimally invasive surgical (MIS) tools, demonstrating high precision on curved surfaces, as well as graphene-integrated optical waveguide devices that dynamically transduce mechanical pressure with robust performance. A notable vision-based advance has been the TacLINK system [7], using stereo cameras to monitor skin deformation across robotic limbs, underscoring the field’s drive toward versatile, geometry-independent tactile integration.

After 2020, the field of tactile sensing has seen further advancements, including the emergence of more compact, energy-efficient, and application-specific systems tailored for next-generation robotics and wearable technologies. For example, Cao et al. [8] presented a polymer-based triaxial sensor on curved shells, measuring both normal and shear forces. Yoo and Yuan [9] demonstrated the use of a vision-embedded GelSight-type sensor for in-hand pose estimation in electronics assembly. Multimodal and transparent systems were proposed by Wang K. et al. [10], combining electrical and optical pathways for self-powered proximity and sliding detection. Wang H. et al. [11] introduced an optical fiber ring resonator for Braille reading, emphasizing fine texture resolution. Hoffmann et al. [12] incorporated neuromorphic processing in NeuroTouch, facilitating real-time gesture recognition with soft optical skins. Yao [13] and Lyu et al. [14] provided a comprehensive review of fiber-optic tactile systems, focusing on their adaptability and precision in robotic and medical devices. Recent work of Yamamoto et al. [15] has demonstrated optical tactile sensors based on self-healing materials and waveguides, where contact-induced deformation disrupts TIR and enables force estimation with reduced sensitivity to electromagnetic noise. For compact integration, Xu et al. [16] introduced ThinTact, a lensless, high-resolution vision-based tactile sensor ideal for grippers and prosthetics. Agarwal et al. [17] used physically based rendering to optimize material-light interactions in optical sensor design, enhancing realism and performance prediction. Do et al. [18] developed DenseTact 2.0, using fisheye optics and elastomer covers for deep learning-based shape and force reconstruction, with future improvements expected from expanded LED arrays and training datasets.

Significant advancement has been achieved in MIS as well, where the loss of haptic feedback—especially in robot-assisted procedures—can compromise surgical precision and patient safety. Conventional electrical sensors, such as piezoelectric or resistive types, have been widely explored, but they face limitations in MRI environments, static load detection, and integration on complex 3D surfaces. Optical tactile sensing has emerged as a promising alternative due to its immunity to electromagnetic interference, compatibility with medical imaging, and potential for miniaturization [19,20]. Recent developments have introduced compact optical waveguide-based sensors capable of conforming to curved surgical instruments, offering high resolution, low hysteresis, and repeatability—key features for accurate force detection in MIS [21]. Systematic reviews have also highlighted the increasing focus on optical methods in robotic endoscopy, particularly for enabling multi-point force sensing in narrow anatomical environments [19]. Emerging designs like the MiniTac sensor integrate mechanoresponsive photonic elastomers with embedded cameras to detect contact forces and even sub-surface structures such as tumors, providing tactile functionality in tools like the Da Vinci surgical system [22]. In parallel, neuromorphic optical sensors such as NeuroTac mimic human skin mechanics and use event-based vision to classify textures, showing promise for tactile perception in both medical and prosthetic applications [23].

Together, these advances underscore the growing relevance of optical tactile sensing in medical robotics, marking a shift from rigid waveguides and fiber optics to precise, flexible, and adaptive multimodal systems with embedded computation. While the field is now poised for broader deployment in surgical tools, soft robotics, wearable technologies, and autonomous interaction platforms, challenges related to scalability and cost efficiency still need to be addressed for widespread adoption.

Traditional frustrated total internal reflection (FTIR)-based touch sensing has been widely utilized in various interactive technologies due to its robust optical detection mechanism and scalability. However, despite its advantages in multi-touch applications, FTIR technology presents fundamental limitations that restrict its adaptability for more advanced robotic and flexible sensing applications. The method relies on infrared (IR) light sources, which are injected into an optically transparent medium and internally reflected until contact with an object, such as a fingertip, disrupts the reflection and scatters the light. This scattered light is then captured by IR cameras positioned beneath or beside the touch surface. While this mechanism allows for precise touch detection, the dependence on multiple IR sources, precise alignment, and high-resolution cameras introduces design and operational constraints, particularly in dynamic environments and in applications on non-planar surfaces. For example, Sinan Alsheikh [24] analyzed FTIR camera-based multi-touch systems, reporting that real-time touch tracking is hindered by the need for high-resolution imaging and computationally intensive signal processing, which can introduce unwanted latency in interactive applications. Similarly, Walker provided a comprehensive review of contact-sensing technologies [25], illustrating that FTIR-based touch sensors require precise calibration of both infrared emitters and cameras, leading to increased complexity and reduced adaptability in applications beyond traditional touchscreen interfaces. Wattanaparinton & Takemura explored vision-based FTIR tactile sensing [26,27], demonstrating that camera placement and infrared source alignment significantly influence detection accuracy. The study emphasized that FTIR sensors struggle with detecting contact on curved or robotic surfaces, making them unsuitable for non-planar applications. The lack of adaptability in flexible or irregularly shaped robotic skins further underscores FTIR’s limitations for next-generation human–robot interaction applications. Additionally, Tompkins [28] investigated alternative optical imaging techniques, noting that FTIR’s sensitivity to surface deformations and material imperfections reduces its effectiveness in non-uniform, dynamic sensing environments, a critical drawback in robotic applications requiring real-time touch sensing [28]. Beyond spatial limitations, FTIR also suffers from technical challenges related to infrared detection and processing latency. Similarly, Fan & Xiao [29] explored latency issues in FTIR touch tracking, demonstrating that synchronizing infrared sources with camera processing algorithms remains a persistent challenge, making it difficult to integrate FTIR into fast-response robotic and tactile systems.

Despite the potential of FTIR-based tactile systems, their limitations in real-time response, scalability, and curved surface integration have become apparent. Consequently, there is an increasing need for alternative FTIR optical tactile sensing technologies, such as direct time-of-flight (ToF) LiDAR-based sensing (e.g., recently developed by the authors of this work [30]), which offers greater flexibility, faster response times, and much cheaper sensor production and integration costs and is adaptable for large area sensorization and to complex robotic surfaces. However, the effectiveness of ToF-based optical tactile sensors nevertheless heavily depends on the material properties of the sensing volume and surface. Materials with high optical transparency and low scattering enhance direct touch detection by maximizing light propagation; however, they offer limited signal return in near-proximity interactions, making them more reliant on high-quality surface contact to ensure reliable sensing. Conversely, materials with excessive scattering can degrade touch accuracy while enhancing near-proximity detection. Striking the right balance between transparency, scattering, and diffuse reflectance is crucial for optimizing ToF-based touch sensors.

### 1.2. Objective and Scope of the Present Study

In this work, we investigate how the interplay of light transmission and scattering behavior in waveguide materials affects the performance of direct ToF-based tactile sensors. Time-resolved ToF signal responses and signal-to-noise characteristics are analyzed during contact with target materials—specifically transparent silicone and plastics in white, gray, and black colors. This evaluation highlights how varying optical properties of the waveguide, in combination with target materials offering either reliable contact (e.g., silicone) or less consistent contact (e.g., plastic), impact sensing performance. The resulting insights guide material selection and design strategies for developing high-performance, cost-effective, and geometry-independent tactile sensing platforms tailored for next-generation human–machine interfaces.

## 2. Materials and Methods

### 2.1. Sample Preparation

Materials were manufactured by different processes: casting in shape for soft material fabrication, 3D printing of various photopolymers, and spin coating accompanied by UV photopolymerization. Commercial acrylic glass samples were used in order to study materials with very low light scattering, μ_s_ < 0.1 cm^−1^, and diffuse reflectance, *R*_d_ < 0.5%. The difference in materials and fabrication processes permitted us to obtain sample of a broad range of μ_s_ and diffuse reflectance *R*_d_ values.

Mechanically elastic silicons, TFC4190 Type 19 and Crystalflex Platinum, were made by mixing the two-component silicone and pouring it into a flat mold. Custom 3D-printed samples were modeled in 3D design software (Blender 3D, version 4.4.3; EasyEda (Standard) v6.5.46; FreeCad v1.0.1) and manufactured using stereolithography and liquid-crystal display (LCD) technologies.

The Elegoo Saturn 2 LCD-3D printer (ELEGOO, Shenchen, China) [31] uses a display matrix with back collimated illumination consisting of a 28 LED matrix emitting UV light at 405 nm to induce masked photocuring and to fabricate samples of MonoCure3D Pro Crystal Clear, TechClear 6123, and Liqcreate—Clear Impact photopolymer resins. The 10-inch LCD had a resolution of 8 k pixel (7680 × 4320) enabling a xy-resolution of 28.5 µm and a z-resolution of 25 µm.

The Formlabs 3D stereolithography printer (Formlabs Inc., Somerville, MA, USA) [32] was used to produce samples of JLC and FormLabs Clear RS-F2-GPCL-04 photopolymer resins. The Formlabs Form 3B+ 3D printer utilizes a precision 405 nm wavelength laser with a power output of 250 mW, integrated within a certified Class 1 Laser Product (EN 60825-1:2007, [33]) Light Processing Unit (LPU). This system employs a laser spot size of 85 µm, enabling high-resolution fabrication with the layer thicknesses ranging from 25 to 300 µm, and operates on low-force stereolithography technology, which minimizes peel forces during the printing process. The samples were printed at its highest resolution of 25 µm at a printing speed of approximately 40–60 layers per hour. Following the printing process, the resin underwent UV curing at a controlled temperature of 60 °C to ensure optimal hardness and durability.

Multilayer samples of FormLabs Clear RS-F2-GPCL-04 resin were produced using a multilayer spin-coating process. First a 125 × 125 mm glass substrate was prepared by rinsing it in acetone, detergent, and isopropanol. This was essential to ensure good adhesion of the first material layer to the substrate. Layer fabrication consisted of a spin-coating step and exposure to a 365 nm UV diode. The first layer was produced by spin-coating the material at 300 rpm to produce a thin but uniform layer of material, with exposure for 30 s. All consecutive layers were spin-coated at 150 rpm and exposed for 30 s as well. Single-layer thickness was estimated to be around 120–150 µm. After the last spin-coating step, the material was removed from the glass substrate using tweezers and rinsed in water to remove any non-crosslinked polymer material.

The list of studied samples—including their names, fabrication methods, geometries and sizes, visual quality assessments, and corresponding photographs—is summarized in Table S1 of the Supplementary Data File.

All experiments described below were conducted under standard ambient conditions (room temperature at ~22 °C and a stable humidity of ~50%), reflecting the typical environments of human–robot collaboration in industrial settings such as automotive manufacturing and packaging. Since temperature and humidity remain relatively constant in such applications, and tactile sensors are not intended to operate near the glass transition range of common polymers, environmental stress testing was considered beyond the scope of this study.

### 2.2. Optical Characterization

The investigation of light scattering properties was carried out using an advanced spectroscopic setup that included the Cary 7000 spectrophotometer, a universal measurement accessory (UMA), and a diffuse reflectance accessory (DRA). The methodological approach implemented in this study is based on the work of F. Foschum et al., which provides a systematic framework for characterizing optical scattering in complex media [34,35].

To accurately determine the scattering characteristics, transmittance *T* and reflectance *R* measurements were performed using the Agilent Cary 7000 equipped (Agilent Technologies, Inc., Santa Clara, CA, USA) with the DRA module. Both specular and diffuse reflectance *R*_d_ components were captured at least at three different places of the sample, allowing a comprehensive assessment of light interaction with the material surface and subsurface. The optical anisotropy factor *g*, which describes the preferential scattering direction, was derived from angularly resolved reflectance *R*_a_ and transmittance *T*_a_ measurements at different detector angles using Cary 7000 equipped with UMA. With these fundamental measurements, the absorption *μ_a_* and scattering *μ*_s_ coefficients were preliminarily estimated based on the experimental reflectance and transmittance data, applying analytical or empirical models for light propagation.

To refine these estimated optical parameters, Monte Carlo Multi-Layered (MCML) simulations were conducted [36]. This simulation method models light propagation within the material, generating theoretical transmittance and reflectance coefficients under the same conditions as those used in the experimental setup. The simulated values for transmittance and reflectance were compared against the experimental measurements. If discrepancies were identified, the optical parameters, including absorption, scattering, and anisotropy factor, were iteratively adjusted, and the simulation was repeated until a satisfactory agreement between measured and simulated values was obtained.

The total hemispherical reflectance *R* and transmittance *T* were then calculated using the following equations:

(1) $$R=\frac{S_{RB}C_{NB}^{R}}{S_{NB}C_{RB}^{R}}\rho_{cal}\;\mathrm{and}$$

(2) $$T=\frac{S_{TB}C_{NB}^{T}}{S_{NB}C_{TB}^{T}},$$

where $S_{RB}$ and $S_{NB}$ represent the measured reflectance signals for the reference and sample, respectively, while $C_{NB}^{R}$ and $C_{RB}^{R}$ are calibration factors associated with the reflectance standard. Similarly, $S_{TB}$ and $S_{NB}$ represent the transmittance signals for the reference and sample, and $C_{NB}^{T}$ and $C_{TB}^{T}$ are calibration factors related to transmittance. The term $\rho_{cal}$ is the known reflectance coefficient of the calibration standard at the corresponding wavelength.

The process involves three key steps: (1) estimating the absorption and scattering coefficient values, (2) applying the MCML model to simulate light propagation within the material and determine the *R* and *T*, and (3) iteratively comparing the simulated *R*_s_ and *T*_s_ values with the measured *R*_e_ and *T*_e_ data until a satisfactory level of agreement is achieved.

The observed local variations in measured transmittance ranged from 0.6% to 2.5%, while reflectance variations were between 0.3% and 0.8%. A high standard deviation in transmittance (~2.5%) and reflectance (~0.8%) was observed for the sample exhibiting strong light scattering, characterized by a scattering coefficient greater than 0.2 cm^−1^. The estimated accuracy in the derived optical parameters is approximately ±0.05 cm^−1^ for the scattering coefficient and ±0.04 for the anisotropy factor across all evaluated samples.

The refractive index of the opaque samples was determined using the Metricon system, which measures the refractive index at specific angles by analyzing the critical coupling angle at three different wavelengths *λ*: 532 nm, 632.8 nm, and 1064 nm. To obtain the spectral dispersion for each material, the experimentally measured values were fitted using Cauchy’s semi-empirical equation, given by

*n* = *A* + *B*/*λ*^2^ + *C*/*λ*^4^*,* (3)

where *A*, *B*, and *C* are material-dependent coefficients.

The refractive index of smooth-surfaced samples, such as acrylic glass, was measured using a variable-angle spectroscopic ellipsometer (RC2-XI, J.A. Woollam Co., Lincoln, NE, USA), operating over a broad spectral range of 210 to 1690 nm (0.7 to 5.9 eV). The resulting ellipsometric spectra were analyzed using CompleteEASE^®^ software (version 6.73), employing model-based regression techniques to achieve accurate fitting of the experimental data. The measurement protocol and analytical approach closely followed the methodology detailed in our recent publication [37], particularly the Supplementary Information of this artilce, which outlines the ellipsometric characterization of soda-lime glass substrates in high precision. The error estimation of refractive index for Metricon system and ellipsometer is ±0.002.

The determination of the refractive index is necessary for anisotropy factor *g* estimation and for contact point calculation on the surface of the light guided material [30]. The refractive index *n* values at 1064 nm, 632.8 nm, and 532 nm, along with the corresponding Cauchy fitting parameters for all samples, are summarized in Table S2 of the Supplementary Data File. To the best of the authors’ knowledge, there is currently no comprehensive study available reporting *n* values for most of the studied 3D-printed materials, including MonoCure3D, Tech-Clear, Liqcreate, and FormLabs resins.

### 2.3. Measurements with ToF

The TMF8828 time-of-flight (ToF) sensor from AMS-OSRAM has been identified as compatible with the research requirements, making it an optimal choice for precise tactile sensing applications as demonstrated in our previous work [30]. Its selection underscores the importance of choosing components that align with the operational parameters necessary to minimize energy losses and maximize the fidelity of captured tactile information.

For data readout and configuration, the TMF8828 utilizes the I^2^C communication protocol, operating at clock speeds of up to 1 MHz. To manage data acquisition and preprocessing, an ESP32-S3 MCU was selected as the communication host, capable of supporting up to four ToF sensors. Each sensor is mounted on an independent PCB and connected to the host MCU via a flexible printed circuit (FPC), allowing for sensor repositioning along the material’s edge to achieve optimal optical coupling.

**Figure 1:**
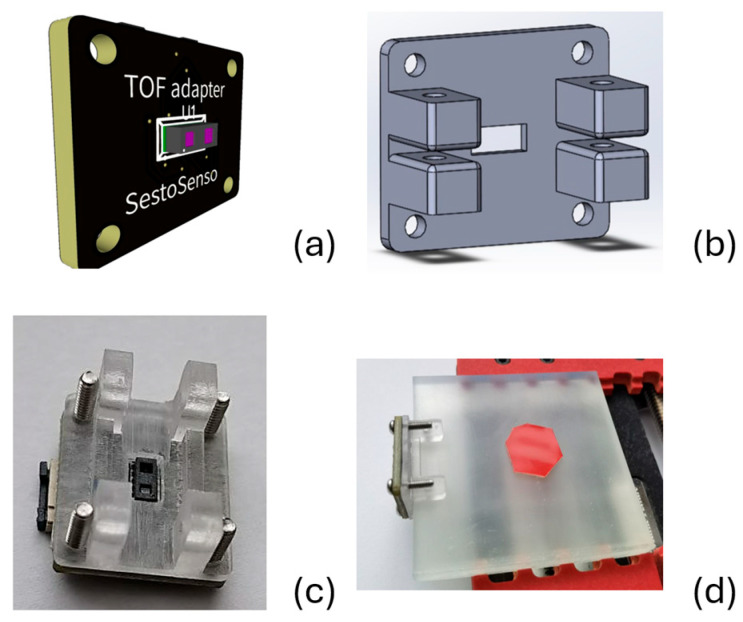
3D models and real-life implementation of a TMF8828-based ToF sensor setup. (**a**) 3D model of the TMF8828 hosting PCB. (**b**) Edge adapter designed for precise positioning of the ToF sensor. (**c**) Actual assembly of the sensor with the mounting adapter. (**d**) Measurement setup, where the ToF sensor is attached to the edge of a polymer test sample with a silicon tape applied to its surface (marked by the red dot).

To ensure the precise alignment of each sensor’s optical axis with the sample’s edge, 3D-printed adapters are used, as illustrated in Figure 1. These adapters are custom-designed to match the specific thickness of the samples, enabling accurate sensor positioning for optimal coupling with the test material.

The first set of experiments was conducted to study the response levels from different materials when a silicone test object was in direct contact with the surface of the experimental polymer. For each sample, the center of the silicone object was positioned 30 mm away from the attached ToF sensor, as shown in Figure 1d. To investigate the potential near-proximity effect, test objects made of glossy 20 × 20 mm plastic of three different colors (white, gray, and black) were used, all having the same size and positioned identically to the silicone object. A separate category included optical-grade soft silicone materials, which not only provided a detection point on the surface but also exerted a push force. For soft material testing, a round steel bar with an 18 mm diameter, 50 mm height, and a weight of 100 g was used as the test object, providing constant pressure. In parallel with the silicone test object, which establishes good contact with the surface, three plastic samples of the same size but in different colors (white, gray, and black) were used to study the so-called near-proximity effect. This effect may result from light leakage caused by structural defects in the printed materials, which act as light-scattering elements.

Unlike the silicone test object, the plastic samples make contact with the surface only at a few points, preventing the observation of signals caused by FTIR. This experiment helps assess the extent of the near-proximity effect for each type of printed material.

## 3. Results and Discussion

### 3.1. Optical Properties

**Figure 2:**
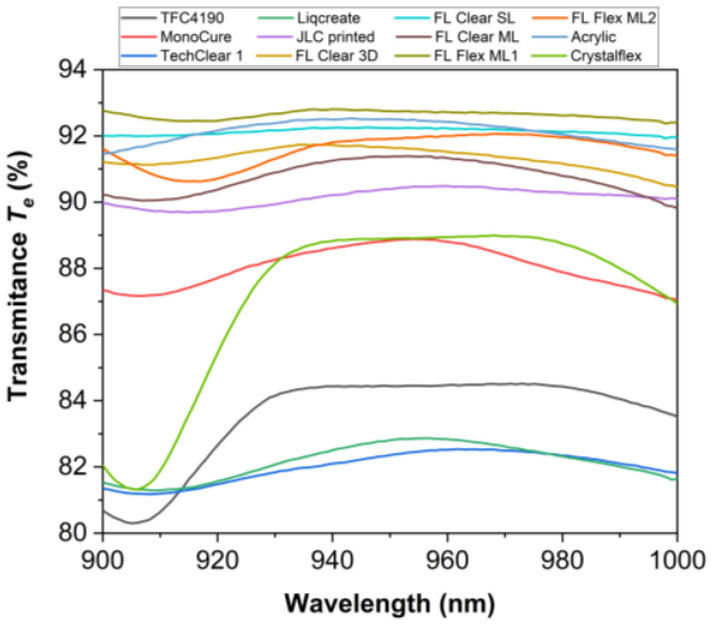
The average experimentally obtained transmittance *T*_e_ of the samples studied.

**Figure 3:**
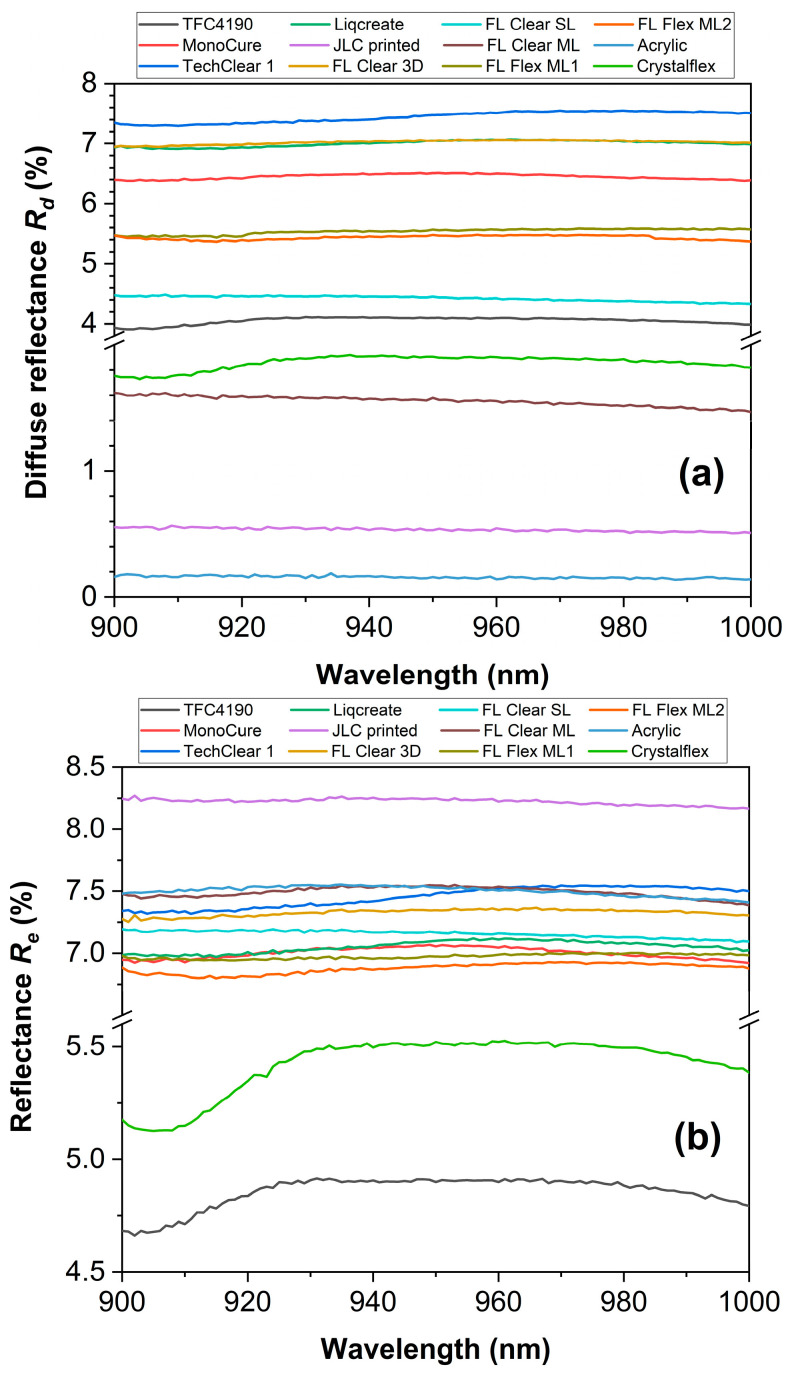
The average (**a**) diffuse reflectance *R*_d_ and (**b**) reflectance *R*_e_ (the sum of diffuse and specular reflectance) values of the samples studied.

Figure 2 and Figure 3 present a comprehensive optical characterization of twelve materials for use in ToF optical skin applications in the near-infrared (NIR) range (900–1000 nm). The investigated parameters include average experimentally measured transmittance *T*_e_ (Figure 2), diffuse reflectance *R*_d_ (Figure 3a), and reflectance *R*_e_ (Figure 3b), providing insights into the light propagation behavior of these materials. Materials such as FL Flex ML, ML1 and ML2, FL Clear 3D, and acryl exhibited the highest transmittance values (>91%), indicating superior transparency and minimal absorption (Figure 2). MonoCure and Crystalflex also showed relatively moderate transmittance, around 88% at 940 nm. In contrast, materials like TechClear 1 and Liqcreate demonstrated the lowest transmittance (~82%), suggesting greater scattering. The *R*_d_ measurements showed significant differences in surface scattering characteristics (Figure 3a). TechClear 1, Liqcreate, MonoCure and FL Clear 3D presented higher *R*_d_ values, >6%, indicative of surface roughness or bulk heterogeneities. Conversely, Acrylic and JLC printed materials exhibited minimal *R*_d_ values, <1%, suggesting optically smoother surfaces and lower scattering. Figure 3b combines both specular and diffuse reflectance components to provide total reflectance *R*_e_ data. Materials such as JLC printed and Acrylic materials showed the highest *R*_e_ values, >7.5, while TFC4190 and Crystalflex exhibited *R*_e_ ~4.5–5.5. Although the materials exhibit relatively high transmittance, their significant diffuse reflectance may adversely affect ToF system performance by contributing to signal artifacts.

**Figure 4:**
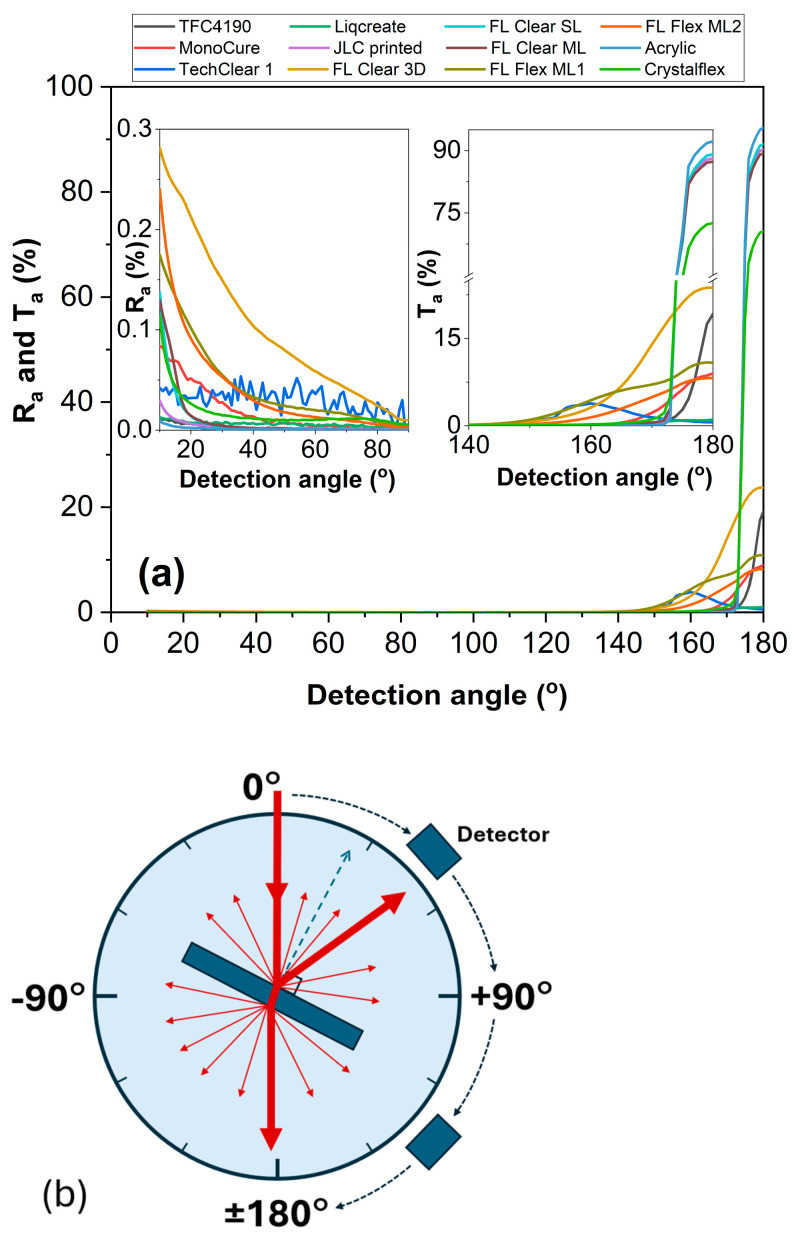
The angle-resolved reflectance *R*_a_ and transmittance *T*_a_ of the samples studied: (**a**) measured *R*_a_ and *T*_a_ as a function of detection angle; and an (**b**) illustration of the experimental configuration, where the detection angle is defined as the angle between the detector position and the incident light beam.

The angle-resolved reflectance *R*_a_ and transmittance *T*_a_ of various polymer materials measured using a rotating detector in the Cary 7000 UMA system are presented in Figure 4. The light source was fixed, while the detector angle varied from 10° to 180° with respect to the incident light, covering both reflection and transmission hemispheres (Figure 4b). For detector angles between 10° and 90°, the system captures reflected light *R*_a_, i.e., light scattered or redirected back toward the incident side. For angles between 90° and 180°, the detector measures transmitted light *T*_a_, i.e., light that has passed through the sample and emerges on the opposite side. At low angles (10–40°), reflectance remains relatively low across all materials, typically below 0.5% (Figure 4a). However, some materials like FL Clear 3D and FL Flex ML2 show slightly elevated *R*_a_, indicating surface roughness or internal scattering. At high angles (140–180°), transmittance increases steeply, reaching values close to 90% for optically clear samples such as acryl, JLC printed, FL Clear SL, FL Clear ML, and Crystalflex (Figure 4a). The rise in *T*_a_ at near−180° angles is expected, as this direction aligns with the unscattered forward beam. Samples such as TechClear 1, Liqcreate, MonoCure, FL Flex ML2, and FL Flex ML1 exhibit lower transmittance at high angles, suggesting increased internal scattering, absorption, or inhomogeneity. In contrast, high-clarity materials maintain strong forward transmission with minimal backward reflection, consistent with a low-loss, optically smooth surface. This angular-resolved measurement method is particularly sensitive to subtle differences in optical quality, scattering behavior, and material homogeneity and provides data for determining the optical anisotropy factor *g*, which quantifies the preferential direction of light scattering within the material. The datasets of Figure 2, Figure 3 and Figure 4 are available online [38].

**Figure 5:**
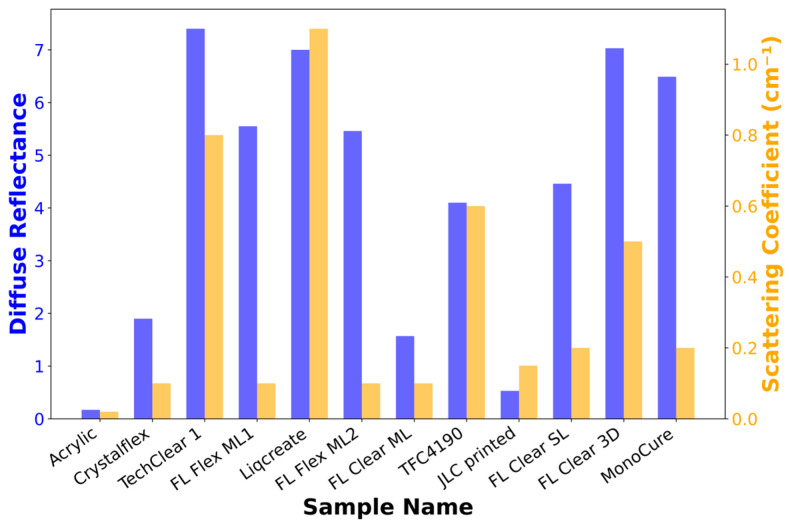
Comparison of diffuse reflectance and scattering coefficient at 940 nm for all test materials.

By systematically analyzing the values of diffuse reflectance *R*_d_, experimental reflectance *R*_e_ and transmittance *T*_e_, and angle-resolved reflectance *R*_a_ and transmittance *T*_a_, the derived optical parameters such as simulated reflectance *R*_s_ and transmittance *T*_s_, scattering coefficient μ_s_, and anisotropy factor *g* were calculated. Table 1 summarizes the obtained optical properties and illustrates the diversity in light propagation behavior across a range of polymeric samples. The comparison of μ_s_ and *R*_d_ between all test samples is presented in this Figure 5. A substantial variation in μ_s_ and reflectance characteristics can be seen, spanning a wide range of *R*_d_, from as low as 0.17% (acrylic glass) to as high as 7.40% (TechClear 6123), and scattering coefficients, from near-zero levels in acrylic glass to over 0.8 cm^−1^ in 3D-printed materials.

Most of the samples show relatively high anisotropy factor *g* values (Table 1), with the exception of TechClear 6123, presenting the lowest value of *g*. The anisotropy factor *g* is a measure of the average direction of scattered light, ranging from −1 (fully backward scattering) to +1 (fully forward scattering). A high anisotropy factor, where here *g* is close to 1, indicates that light, when scattered, predominantly continues in its original direction with minimal angular deviation. Acrylic glass demonstrates an exceptionally high anisotropy factor of 0.998 cm^−1^, meaning that almost all scattered photons are redirected forward with negligible lateral or backward scattering. This is accompanied by a very low scattering coefficient (μ_s_ ≈ 0.02 cm^−1^), which quantifies the frequency of scattering events per unit distance. These optical characteristics arise from the intrinsic material purity, homogeneity, and molecular uniformity of acrylic glass. Its structure lacks the internal irregularities, refractive index fluctuations, or surface roughness that commonly induce light scattering in other materials. As a result, acrylic glass is nearly transparent to light and serves as a reference material in optical experiments, especially where clarity and minimal light diffusion are essential.

Notably, 3D-printed samples such as TechClear 6123 and Liqcreate exhibit elevated scattering coefficients (0.8 cm^−1^ and 1.1 cm^−1^, respectively) and low values of anisotropy factors (0.874 and 0.911 cm^−1^), indicating a dominance of non-forward light scattering. This behavior is consistent with the voxel-based fabrication process intrinsic to 3D printing, where local refractive index fluctuations and pixel-level misalignments increase heterogeneity and photon diffusion. These scattering effects ultimately degrade the material’s optical clarity and limit its utility in high-resolution optical sensing applications.

In contrast, multi-layer spin-coated samples, particularly FormLabs Clear—Multi Layer, exhibit a comparatively lower scattering coefficient (0.1 cm^−1^) and higher anisotropy (*g* = 0.926 ÷ 0.995 cm^−1^), reflecting more forward-directed scattering and improved optical transparency. These results underscore the advantages of layered deposition in minimizing internal optical discontinuities and enhancing uniformity. When comparing the performance between different FormLabs materials, FormLabs Clear consistently outperforms FormLabs Flex in both single- and multi-layer configurations in terms of optical clarity, offering higher transmittance and lower diffuse reflectance.

To the best of the authors’ knowledge, no comprehensive study is available reporting the refractive index or scattering coefficient for the majority of the studied 3D-printed materials, including MonoCure3D, TechClear, Liqcreate, and FormLabs resins. For TFC4190 Type 19 and Crystalflex silicones, however, the experimentally determined refractive index values align well with those reported for low-index silicone systems, typically ranging from 1.40 to 1.45 depending on formulation and curing state. Similarly, the measured refractive index for acrylic glass corresponds closely with values listed in the CompleteEASE^®^ optical database of PMMA, confirming the accuracy of the applied ellipsometric methods.

### 3.2. ToF Measurements

**Figure 6:**
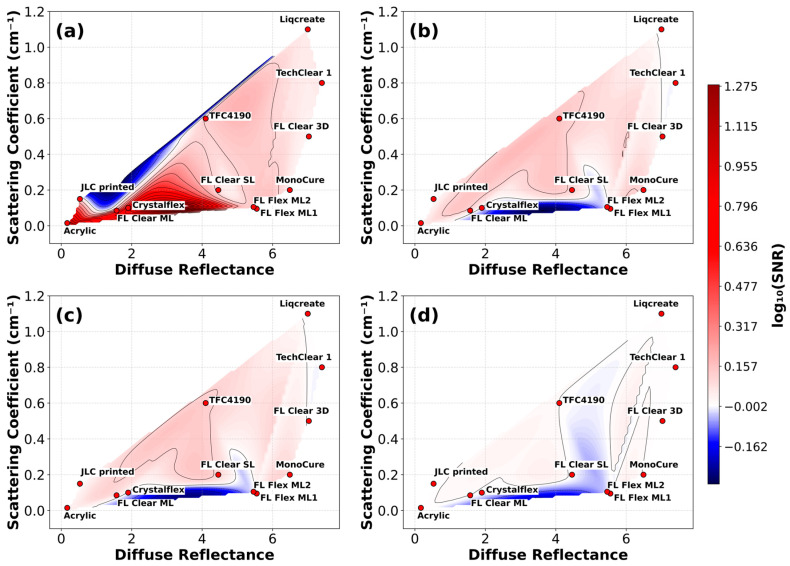
Contour plots of log_10_(SNR) vs. diffuse reflectance and scattering coefficient for various target materials: Figures (**a**–**d**) show the relationship between log_10_(SNR), diffuse reflectance, and scattering coefficient for the silicone, white, gray, and black targets, respectively. Each plot visualizes the optical properties of the materials, where color gradients represent the range of SNR values. Redish colors correspond to higher SNR values, while white and blue colors indicate lower SNR values. An alternative version of this plot using the HSV color model is available in the Supplementary Data File.

The set of graphs in Figure 6 illustrates the relationship between the logarithm of the signal-to-noise ratio log_10_(SNR), diffuse reflectance, and the scattering coefficient (cm^−1^) for four categories of targets in contact with studied materials: silicone, white, gray, and black. Each plot provides a two-dimensional visualization of the optical behavior of various materials, where the *x*-axis represents diffuse reflectance and the *y*-axis represents the scattering coefficient. Overlaid color gradients encode the corresponding log_10_(SNR) values, enabling a comparative assessment of how different combinations of optical properties affect signal quality. Red and pink colors denote regions of higher SNR values, indicative of better contact detection performance, while cooler like white and blue represent lower SNR values, corresponding to poorer signal clarity. Material samples are plotted as red markers with identifying labels positioned adjacent to each point.

For the silicone target, optimal performance is observed in materials exhibiting both low diffuse reflectance and low scattering coefficients. This region, typically situated in the lower-left quadrant of the plot (Figure 6a), is associated with the highest SNR values, implying that transparency and minimal internal scattering are critical for maximizing signal fidelity in silicone-based contact scenarios. Materials such as acrylic, FL Clear ML, and CrystalFlex fall within this favorable zone. In contrast, increasing either reflectance or scattering markedly reduces the SNR, indicating a degradation in optical quality for silicone targets under these conditions.

In the case of white targets (Figure 6b), the highest SNR values are concentrated in regions with moderate-to-high diffuse reflectance combined with increased scattering. This suggests that the visibility of white plastic targets is enhanced when multiple scattering events occur within a highly reflective medium. Materials such as Liqcreate and TechClear 1 exemplify this behavior, occupying the upper-right region of the plot where SNR is maximized (Figure 6b). Conversely, low-scattering and low-reflectance materials, such as FL Flex ML2 and acrylic, demonstrate poor performance, resulting in diminished signal quality.

However, materials exhibiting low scattering and low diffuse reflectance—such as acrylic—typically have low surface roughness, which favors optical clarity but can make them highly sensitive to contact quality. Consequently, achieving a uniform interface, such as between silicone and acrylic, is essential for efficient FTIR signal coupling and high SNR performance. However, when paired with targets like white plastics, which may have rougher or more heterogeneous surfaces, the contact quality at the interface with acryl can deteriorate. This suboptimal physical contact can introduce optical mismatches or air gaps, leading to reduced signal transmission and, therefore, lower SNR values for acrylic in such configurations.

Gray targets (Figure 6c) display a more complex response, with two distinct zones of optimal performance. The first is characterized by high scattering and high diffuse reflectance, where materials like Liqcreate and TechClear 1 again demonstrate elevated SNR values. The second favorable region lies in an intermediate reflectance range (approximately 3%) coupled with low scattering. Here, materials such as FL Clear ML and Crystalflex achieve comparable performance, indicating that gray targets can be effectively detected either through strong scattering contrast or through optically clear media that transmit moderate levels of diffuse light.

For black targets (Figure 6d), the system demonstrates a low degree of robustness across a broad range of material properties. Most materials, irrespective of whether they are highly scattering or minimally scattering, yield very low SNR values. In the region centered around 4.5% diffuse reflectance and low scattering, a notable drop in SNR is observed, as seen in materials such as TFC4190. This suggests that black targets are generally more difficult to detect across varied material profiles under direct ToF measurements at 940 nm wavelength.

**Figure 7:**
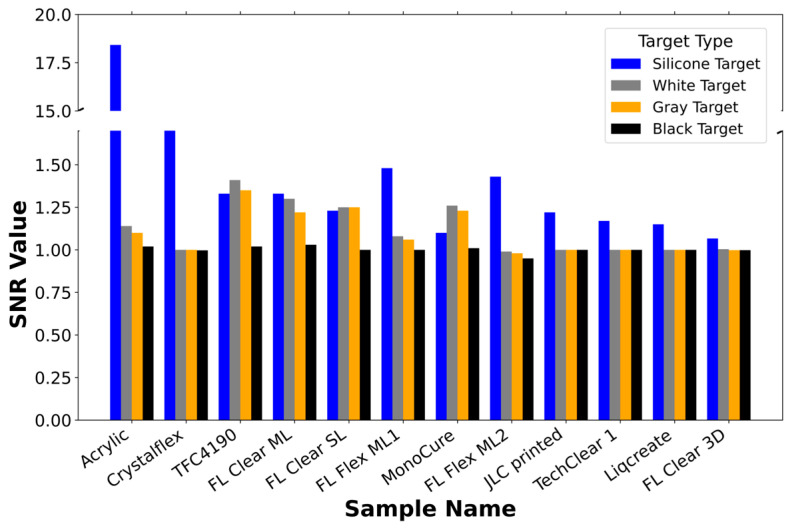
Comparison bar graph of the SNR for different materials when exposed to silicone, white, gray, and black targets. The materials are sorted by their average SNR across all target types, with silicone targets represented in blue, white targets in orange, gray targets in gray, and black targets in black. The SNR values reflect the response of the material to time-of-flight (ToF) measurements, showing the sensitivity of each material to light interaction in both direct contact (FTIR) and near-proximity sensing.

A direct SNR comparison for all materials across target types is given in Figure 7. Sorted by average SNR, it can be seen that materials like acrylic and Crystalflex consistently deliver high SNR values in FTIR conditions (Silicone target). These materials possess smooth, homogeneous surfaces and low scattering coefficients (Figure 4), enabling strong light confinement and coupling to the ToF detector. Commercial acrylic glass, in particular, achieves the highest SNR ≈ 18.42, underscoring the importance of optical clarity and surface quality in maximizing FTIR for tactile sensing. On the other hand, 3D-printed materials such as TechClear 1 and Liqcreate perform poorly in FTIR (silicone contact) scenarios due to excessive internal scattering (Figure 4). Their high scattering coefficients (up to 1.1 cm^−1^) lead to light loss via angular dispersion and disruption of TIR, which diminishes coupling efficiency and SNR. These materials instead rely more heavily on near-proximity sensing, where light leaks out and reflects off nearby surfaces.

## 4. Conclusions

A systematic optical characterization of twelve polymeric materials was conducted to assess their performance in ToF sensing for both direct-contact (FTIR-based) and near-proximity configurations. The results indicate that the control of internal light scattering is a critical factor influencing signal-to-noise ratio (SNR) and detection reliability. A strong dependence was observed between sensor performance and the interplay of diffuse reflectance and scattering coefficient across different background target conditions.

The findings emphasize the necessity of selecting materials with appropriate optical parameters—particularly scattering behavior, refractive index uniformity, and surface finish—to meet the requirements of specific sensing modalities. The analysis further delineates a performance trade-off between FTIR-based and near-proximity detection mechanisms, summarized as follows:

- Low-scattering, optically transparent materials (e.g., acrylic, FL Clear ML) are optimal for FTIR-based contact sensing.
- High-scattering, moderately reflective materials (e.g., TechClear 1, Liqcreate) are more effective for near-proximity sensing.
- Black targets exhibit reduced detectability due to intrinsic optical limitations.

These insights provide a structured framework for the design and selection of materials in ToF-based tactile and proximity sensing systems, enabling more targeted material development and improved system-level integration for a range of optical interface applications.

For robotic sensorization aimed at enhancing environmental perception, material selection should prioritize those capable of reliable contact detection via FTIR and effective proximity sensing through controlled light scattering or engineered microstructured surfaces for directional light management.

## Figures and Tables

**Table 1:** The diffuse reflectance, *R*_d_, scattering coefficient μ_s_, anisotropy factor *g*, simulated reflectance *R*_s_ and experimentally obtained reflectance *R*_e_, simulated transmission *T*_s_ and experimentally obtained transmission *T*_e_, and refractive index at *n*@940 nm for all studied samples.

Sample	*R*_d_, %	μ_s_, cm^−1^	*g*, cm^−1^	*R*_s_, %	*R*_e_, %	*T*_s_, %	*T*_e_, %	*n*
**TFC4190 Type 19** **Sample 1**	4.10	0 * 0.6 **	0.99 * 0.993 **	4.87	4.92	84.58	84.40	1.401
**MonoCure3D Pro Crystal Clear 2**	6.49	0.2	0.931	6.96	7.05	87.88	88.60	1.483
**TechClear 6123** **Sample 1 (TechClear 1)**	7.40	0.8	0.874	7.32	7.4	82.23	82.1	1.537
**Liqcreate—Clear Impact 2**	7.00	1.1	0.911	7.02	7.05	82.54	82.50	1.523
**JLC printed**	0.53	0.15	0.998	8.05	8.25	90.51	90.21	1.519
**FormLabs Clear—3D-printed (FL Clear 3D)**	7.03	0.5	0.940	7.63	7.34	91.01	91.72	1.497
**FormLabs Clear—Single layer (FL Clear SL)**	4.46	0.2	0.996	7.52	7.17	91.78	92.25	1.497
**FormLabs Clear—Multi layer (FL Clear ML)**	1.57	0.1	0.995	7.51	7.53	91.04	91.25	1.497
**FormLabs Flexible—Multi layer 1 (FL Flex ML1)**	5.55	0.1	0.934	7.28	6.96	92.42	92.80	1.482
**FormLabs Flexible—Multi layer 2 (FL Flex ML2)**	5.46	0.1	0.926	7.11	6.87	91.40	91.80	1.482
**Acrylic glass**	0.17	0.02	0.998	7.26	7.54	92.32	92.49	1.483
**Crystalflex**	1.9	0.1	0.995	5.00	5.50	88.06	88.20	1.398

* For glossy area; ** for opaque area.

## Data Availability

The original contributions presented in this study are included in the article/Supplementary Material. Further inquiries can be directed to the corresponding author.

## Supplementary Materials

The following supporting information can be downloaded at: https://www.mdpi.com/article/10.3390/ma18143287/s1, Table S1: The list of the samples studied, including sample name, fabrication method, sample geometry and size, sample visual quality and photo; Table S2: Refractive index n at 1064 nm, 632.8 nm and 532 nm and Cauchy fitting parameters for all samples; Figure S1: Contour plots of log_10_(SNR) vs Diffuse Reflectance and Scattering Coefficient for various target materials: (a–d) show the relationship between log_10_(SNR), diffuse reflectance, and scattering coefficient for the Silicone, White, Gray, and Black targets, respectively. Each plot visualizes the optical properties of the materials, where color gradients represent the range of SNR values. Blue, violet, pink and dark red colors correspond to higher SNR, while light blue, green, yellow and red colors indicate lower SNR values.

## Abbreviations

The following abbreviations are used in this manuscript:

CCD	Charge-coupled device
DRA	Diffuse reflectance accessory
FTIR	Frustrated total internal reflection
HSV	HSV color model (Hue, Saturation, Value)
LiDAR	Light detection and ranging
MCML	Monte Carlo Multi-Layered
MIS	Minimally invasive surgary
MRI	Magnetic resonance imaging
RGB	Red, Green, and Blue (additive color model)
SE	Spectroscopic ellipsometry
ToF	Time-of-flight
TIR	Total internal reflection
UMA	Universal measurement accessory
