# Supplementary material for: Influence of Material Optical Properties in Direct ToF LiDAR Optical Tactile Sensing: Comprehensive Evaluation
Source: Materials (Basel). 2025 Jul 11;18(14):3287. doi: 10.3390/ma18143287 (PMC12299963; doi:10.3390/ma18143287)
Supplement: Supplementary file 1 [file materials-18-03287-s001.zip › materials-3728119-supplementary.pdf]

Supplementary data

All samples were prepared in at least 2-3 exemplars. Optical measurements using a spectrophotometer were performed at multiple locations on each sample to obtain a statistically robust dataset characterizing the optical properties of each material. In contrast, measurements using a prism coupler and ellipsometer were performed at a single location per sample. The refractive index values obtained from 2-3 samples of the same material showed high consistency, with deviations within ±0.002.

Table S1 provides a comprehensive overview of all studied samples, including their names, fabrication methods, geometries, dimensions, visual quality assessments, and representative photographs.

Table S2 presents the measured refractive index *n* values at three wavelengths (1064 nm, 632.8 nm, and 532 nm), along with the corresponding Cauchy fitting parameters for each material. These values were obtained through prism coupling and ellipsometric analyses.

Table S1. The list of the samples studied, including sample name, fabrication method, sample geometry and size, sample visual quality and photo.

Sample/ Material	Fabrication method	Sample geometry, size	Sample quality	Sample photo
**TFC4190 Type 19 Sample 1**	Casting in shape	Disc: 50x8 mm	Glossy, opaque, light scattering	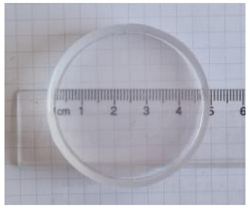
**MonoCure3D Pro Crystal Clear 2**	3D printer, Elegoo Saturn 2	Parallelepiped: 50x50x5mm	Glossy, opaque, light scattering	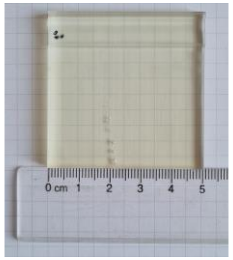

**TechClear 6123 Sample 1**	3D printer, Elegoo Saturn 2	Parallelepiped: 50x50x5mm	Glossy, opaque, light scattering	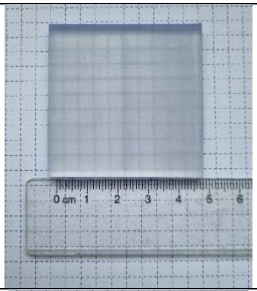
**Liqcreate - Clear Impact 2**	3D printer, Elegoo Saturn 2	Parallelepiped: 50x50x5mm	Glossy, opaque, light scattering	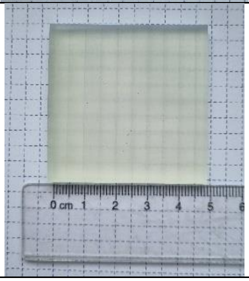
**JLC printed**	3D printer, Elegoo Saturn 2	Parallelepiped: 50x26x1.9mm	Glossy, Transparent	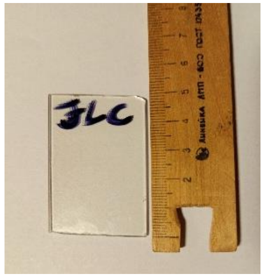
**FormLabs Clear – 3D printed (FL Clear 3D)**	Formlabs 3D printer	Parallelepiped: 135x124x2.6mm	Glossy, light scattering	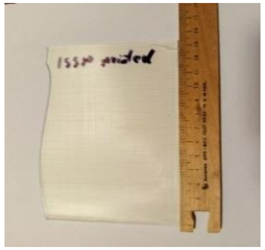

**FormLabs Clear – Single layer (FL Clear SL)**	Spin-coating	Parallelepiped: 75x60x0.6mm	Transparent, homogeneous	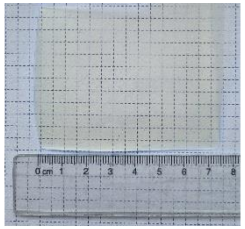
**FormLabs Clear – Multi layer (FL Clear ML)**	Spin-coating	Parallelepiped: 115x125x3.6mm	Transparent, homogeneous	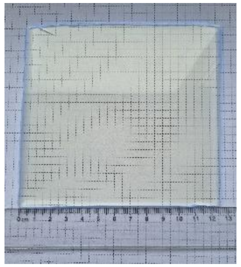
**FormLabs Flexible – Multi layer 1 (FL Flex ML1)**	Spin-coating	Parallelepiped: 50x35x1.2mm	Opaque, light scattering	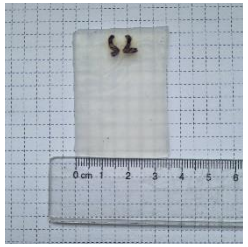
**FormLabs Flexible – Multi layer 2 (FL Flex ML2)**	Spin-coating	Parallelepiped: 55x50x4.2mm	Opaque, light scattering	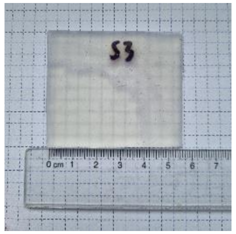

**Acrylic glass**	Commercial	Parallelepiped: 30x30x2mm	Transparent, homogeneous	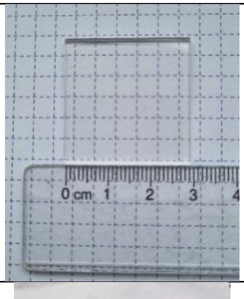
**Crystalflex Platinum 2-component silicone rubber**	Casting in shape	Parallelepiped: 75x85x10mm	Optically clear, fully flexible samples, minimal imperfections (bubbles)	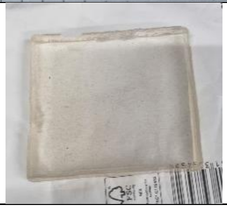

Table S2. Refractive index *n* at 1064 nm, 632.8 nm and 532 nm and Cauchy fitting parameters for all samples.

Sample	*n* at 1064 nm, 632.8 nm and 532 nm	$n = A + B/\lambda^2 + C/\lambda^4$	Measurement method
**TFC4190 Type 19**	1.397@1064 nm<br>1.403@632.8 nm<br>1.408@532 nm	*A* = 1.395635 ± 0.001142<br>*B* = 0.003952 ± 4.04E-05<br>*C* = 0.000103 ± 4.87E-05	Prism method
**MonoCure3D Pro Crystal Clear**	1.482@1064 nm<br>1.490@632.8 nm<br>1.497@532 nm	*A* = 1.47825 ± 0.00013<br>*B* = 0.003376 ± 3.6E-05<br>*C* = 0.000533 ± 1.0E-05	
**TechClear 6123**	1.537@1064 nm<br>1.543@632.8 nm<br>1.556@532 nm	*A* =1.534505 ± 0.001045<br>*B* = 0<br>*C* = 0.001676 ± 3.86E-05	
**Liqcreate**	1.523@1064 nm<br>1.527@632.8 nm<br>1.534@532 nm	*A* = 1.52214 ± 0.00053<br>*B* = 0<br>*C* = 0.000901 ± 4.3E-05	
**JLC printed**	1.519@1064 nm<br>1.529@632.8 nm<br>1.543@532 nm	*A* = 1.51619 ± 0.00065<br>*B* = 0<br>*C* = 0.002028 ± 5.2E-05	
**FormLabs Clear – 3D printed (FL Clear 3D)**	1.497@1064 nm<br>1.5026@632.8 nm<br>1.5107@532 nm	*A* = 1.49580 ± 0.00061<br>*B* = 0<br>C = 0.001173 ± 4.8E-05	
**FormLabs Clear – Single layer (FL Clear SL)**	1.497@1064 nm<br>1.502@632.8 nm<br>1.511@532 nm	*A* = 1.49535 ± 0.00076<br>*B* = 0.000256 ± 2.1E-05<br>*C* = 0.001116 ± 6.1E-05	
**FormLabs Clear – Multi layer (FL Clear ML)**	1.497@1064 nm<br>1.503@632.8 nm<br>1.511@532 nm	*A* = 1.49570 ± 0.00060<br>*B* = 0.000256± 1.7E-05<br>*C* = 0.001103 ± 4.8E-05	

**FormLabs Flexible – Multi layer 1 (FL Flex ML1)**	1.481@1064 nm<br>1.483@632.8 nm<br>1.494@532 nm	*A* = 1.47836 ± 0.00058<br>*B* = 0.00324 ± 0.00016<br>*C* = 0.000358 ± 4.6E-05	
**Crystalflex Platinum 2-component silicone rubber**	1.397@1064 nm<br>1.403@632.8 nm<br>1.408@532 nm	*A* = 1.393856 ± 7.4E-06<br>*B* = 0.002918 ± 2.1E-06<br>*C* = 0.0003413 ± 6.0E-7	
**Acrylic glass (PMMA bulk)**	1.484@1064 nm<br>1.489@632.8 nm<br>1.491@532 nm	*A* = 1.483 ± 2.2420E-05<br>*B* = 0.00212 ± 9.2924E-06<br>*C* = 2.7198E-07 ± 6.4334E-07	Spectroscopic ellipsometry

An alternative version of the Figure 6 using the HSV color model

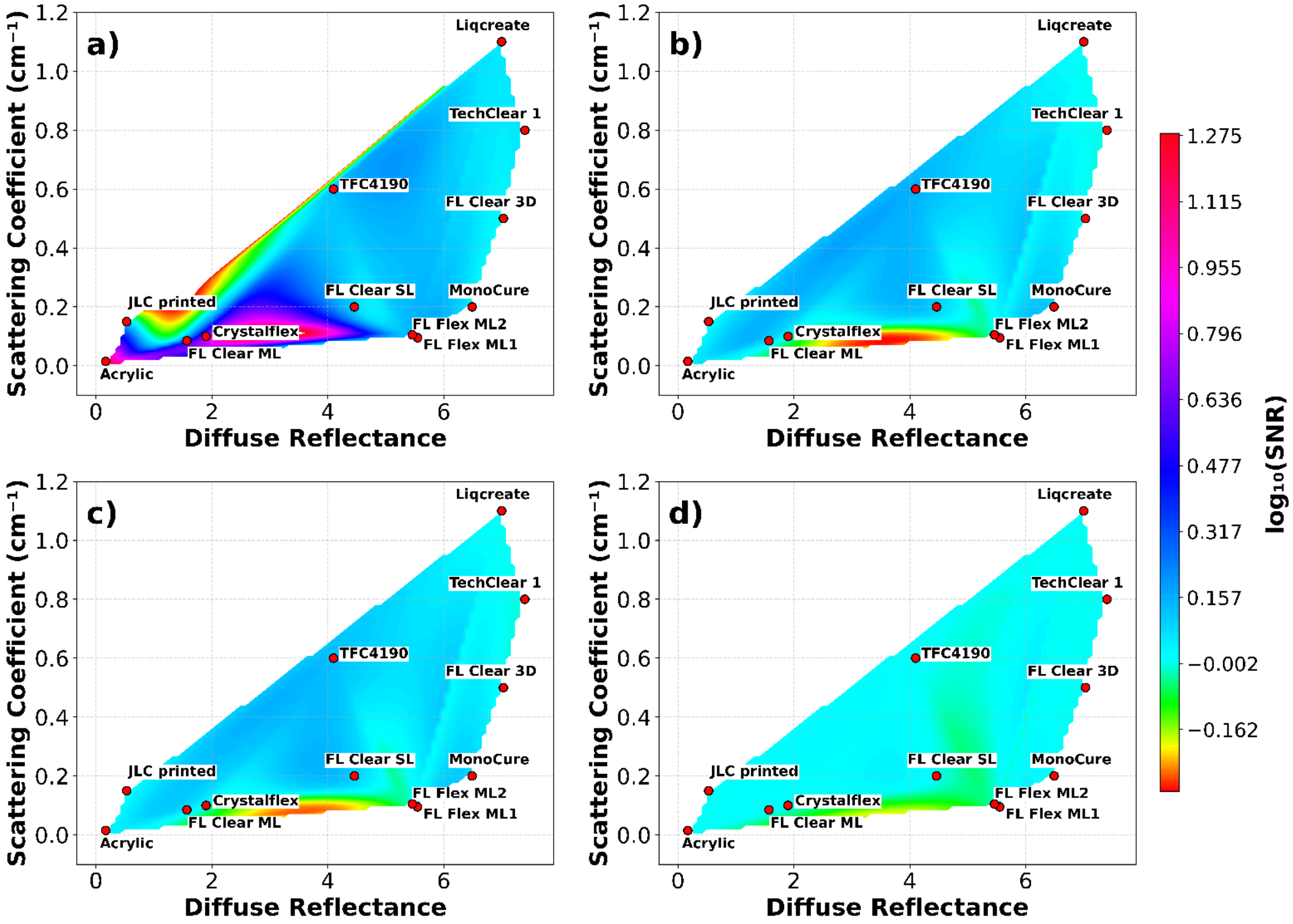

Figure S1. Contour plots of $\log_{10}$(SNR) vs Diffuse Reflectance and Scattering Coefficient for various target materials: (**a–d**) show the relationship between $\log_{10}$(SNR), diffuse reflectance, and scattering coefficient for the Silicone, White, Gray, and Black targets, respectively. Each plot visualizes the optical properties of the materials, where color gradients represent the range of SNR values. Blue, violet, pink and dark red colors correspond to higher SNR, while light blue, green, yellow and red colors indicate lower SNR values.
